# Determining the precise extent of sentinel basins during laparoscopic gastrectomy for early gastric cancer

**DOI:** 10.1186/s12957-023-02953-7

**Published:** 2023-02-23

**Authors:** Sung Eun Oh, Ji Yeong An, Jae-Seok Min, Sang-Ho Jeong, Keun Won Ryu

**Affiliations:** 1 Department of Surgery, Samsung Medical Center, Sungkyunkwan University School of Medicine, Seoul, Republic of Korea; 2 Department of Surgery, Dongnam Institute of Radiological and Medical Sciences, Cancer Center, Busan, Republic of Korea; 3 Department of Surgery, Gyeongsang National University College of Medicine and Gyeongsang National University Changwon Hospital, Changwon, Republic of Korea; 4 Center of Gastric Cancer, National Cancer Center, Goyang, Republic of Korea

**Keywords:** Sentinel basin, Dissection, Laparoscopy, Stomach preserving surgery, Early gastric cancer

## Abstract

**Purpose:**

By analyzing sentinel basin dissection (SBD) data from the SEntinel Node ORIented Tailored Approach (SENORITA) trial, we sought to determine the precise extent of the sentinel basin (SB) without a tracer.

**Materials and methods:**

This study investigated SB length in patients (*n* = 25) who underwent laparoscopic SBD for early gastric cancer (EGC) in the SENORITA trial. SB length along the greater curvature (GC) and lesser curvature (LC) was measured intraoperatively before performing SBD.

**Results:**

In all 25 cases, along the LC of the stomach, the lengths of the SB were 3.7 cm [2.0–5.0] (median [min–max]) proximally and 3.0 cm [2.3–5.5] distally; along the GC side, the lengths of the SB were 6.8 cm [3.5–11.0] proximally and 7.0 cm [3.8–9.5] distally from the tumors. The SB length at the GC or LC side was not significantly different between subgroups categorized by tumor depth, size, and longitudinal location. When tumors were located at the anterior wall of the stomach, the length of the proximal SB (10.0 cm [9.0–11.0]) at the GC side was the longest. In cases with several sentinel lymph nodes (SLNs), the lengths of the SB at the GC side were significantly longer than those with fewer SLNs. However, the lengths of the SB were similar on the LC side regardless of the number of SLNs.

**Conclusions:**

This pilot study had some limitations of a small number of enrolled patients, the lack of research on the specific station of SLNs, and the inaccurate indication for sentinel node navigation surgery (SNNS) without tracer. Nevertheless, the present study which reported the extents of SBs might be the first step towards simplifying procedures in laparoscopic SNNS for stomach preservation in EGC.

## Introduction

Due to well-organized cancer screening programs, the rate of early gastric cancer (EGC) detection is increasing, especially in Korea [1, 2]. EGC is unlikely to recur after gastrectomy; therefore, improving the quality of life of long-term survivors has become a priority. Recently, minimally invasive surgery, including laparoscopic surgery, has been used to improve the quality of life of patients with EGC. Laparoscopic surgery has the advantage of fewer postoperative complications, especially wound complications, and faster surgical recovery compared to open surgery [3]. However, with any surgical approach, gastrointestinal surgeons perform subtotal or total gastrectomy with D1 + lymph node dissection in the treatment of EGC [4, 5].

Recently, results have been reported from a prospective randomized controlled trial (RCT) of laparoscopic sentinel node navigation surgery (SNNS) for stomach preservation in patients with EGC [6, 7]. The patients participating in the clinical trial were diagnosed with stage IA gastric adenocarcinoma (less than 3 cm in size) before surgery; after randomization, patients assigned to a stomach preserving surgery group underwent sentinel basin dissection (SBD) [8]. When there were no metastatic lymph nodes in the sentinel basin (SB) as confirmed by intraoperative frozen section biopsy, the stomach preservation procedure, such as endoscopic submucosal dissection, wedge resection, or segmental resection, was performed instead of conventional radical gastrectomy with D1 + lymph node dissection. However, stomach preserving surgery is more complicated than conventional radical gastrectomy. The operative procedures for stomach preservation are complex, with many steps including endoscopic tracer injection and detection. More preparations are needed for stomach preserving surgery compared to conventional gastrectomy. If the extent of the SB during surgery could be easily identified, laparoscopic stomach preserving surgery would become a simpler procedure and be easier for surgeons to perform.

Through SBD analysis of the SEntinel Node ORIented Tailored Approach (SENORITA) trial, we aimed to investigate the precise extent of sentinel basins without a tracer during laparoscopic gastrectomy for EGC [6, 7].

## Materials and methods

This study investigated patients (*n* = 25) who were enrolled in the prospective SENORITA trial and randomized to the experimental arm, in which the patients underwent laparoscopic SBD. The SENORITA trial was an investigator-initiated, open-label, parallel-assigned, multicenter phase III RCT. The trial assessed the efficacy and safety of laparoscopic SNNS with stomach preservation compared with laparoscopic standard gastrectomy with lymphadenectomy in EGC that was identified between March 2013 and December 2016 [6, 8]. Based on the protocol, the SENORITA trial included patients with clinical T1N0M0 gastric adenocarcinoma, a gastric tumor smaller than 3 cm as the longest diameter, or a gastric tumor at least 2 cm from the pylorus or cardia [8]. All 25 patients enrolled in the present study underwent laparoscopic SBD at the Dongnam Institute of Radiological and Medical Sciences (DIRAMS) Cancer Center, which was qualified to participate in the phase III trial following completion of a prior quality control study [9, 10]. Among the institutions participating in the SENORITA trial, DIRAMS Cancer Center was the only one that assessed the lengths of the SB during SNNS. The primary outcome of this study was length of the SB along the greater curvature and lesser curvature according to tumor characteristics.

### Patient data

Clinicopathologic characteristics of age, sex, body mass index, operation time, sentinel study time, operation type, tumor size, pathologic stage, number of sentinel lymph nodes (SLNs), and length of the SB were ascertained from medical records collected for the SENORITA trial [6]. Sentinel study time is the time from the first injection of tracer to identification of the frozen biopsy result for the SLNs. The pathologic stage was classified according to the eighth edition of the American Joint Committee on Cancer Classification [11]. We also surveyed recurrence of the 25 patients enrolled from the prospectively collected clinical data in DIRAMS. This study was approved by the Institutional Review Board (IRB) of DIRAMS Cancer Center in Republic of Korea (IRB number D-1304–002-001).

### Measurement of sentinel basin length

**Fig. 1:**
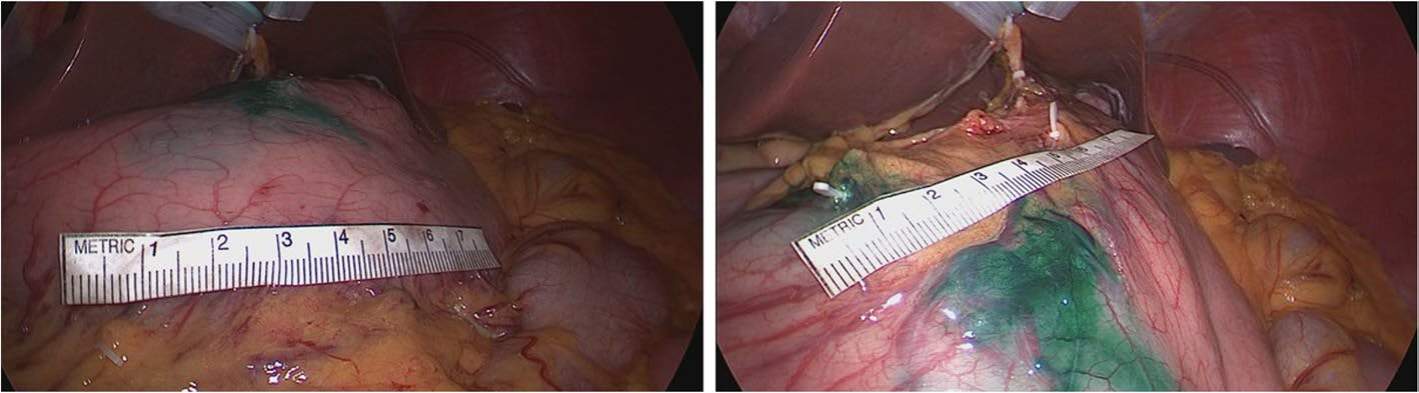
Lengths of the sentinel basin of the greater curvature side (left) and lesser curvature side (right) according to tumor location before laparoscopic sentinel basin dissection

A detailed description of the SBD procedure is provided in a previous report on a quality control study for the SENORITA trial [9, 10]. Briefly, a combination of indocyanine green (ICG; Diagnogreen®, Daiichi-Sankyo Co., Ltd., Japan; 2 mL, 5 mg) and radiolabeled human serum albumin (Tc99m-HSA; 2 mL, 0.1 mCi/mL) was used as the tracer to detect SBs. A 4-mL volume of the dual tracer was injected into the submucosal layer in four quadrants of the primary tumor via an intraoperative endoscopic approach. At 15 min after endoscopic tracer injection, the extent of the SBDs was identified grossly and by laparoscopic handheld gamma probe. The proximal and distal margins of SBDs were marked by laparoscopic surgical clips. Before performing the SBD procedure, the lengths of the SBs were measured using a laparoscopic ruler in the abdominal cavity at the lesser curvature and greater curvature sides of the stomach (Fig. 1).

**Fig. 2:**
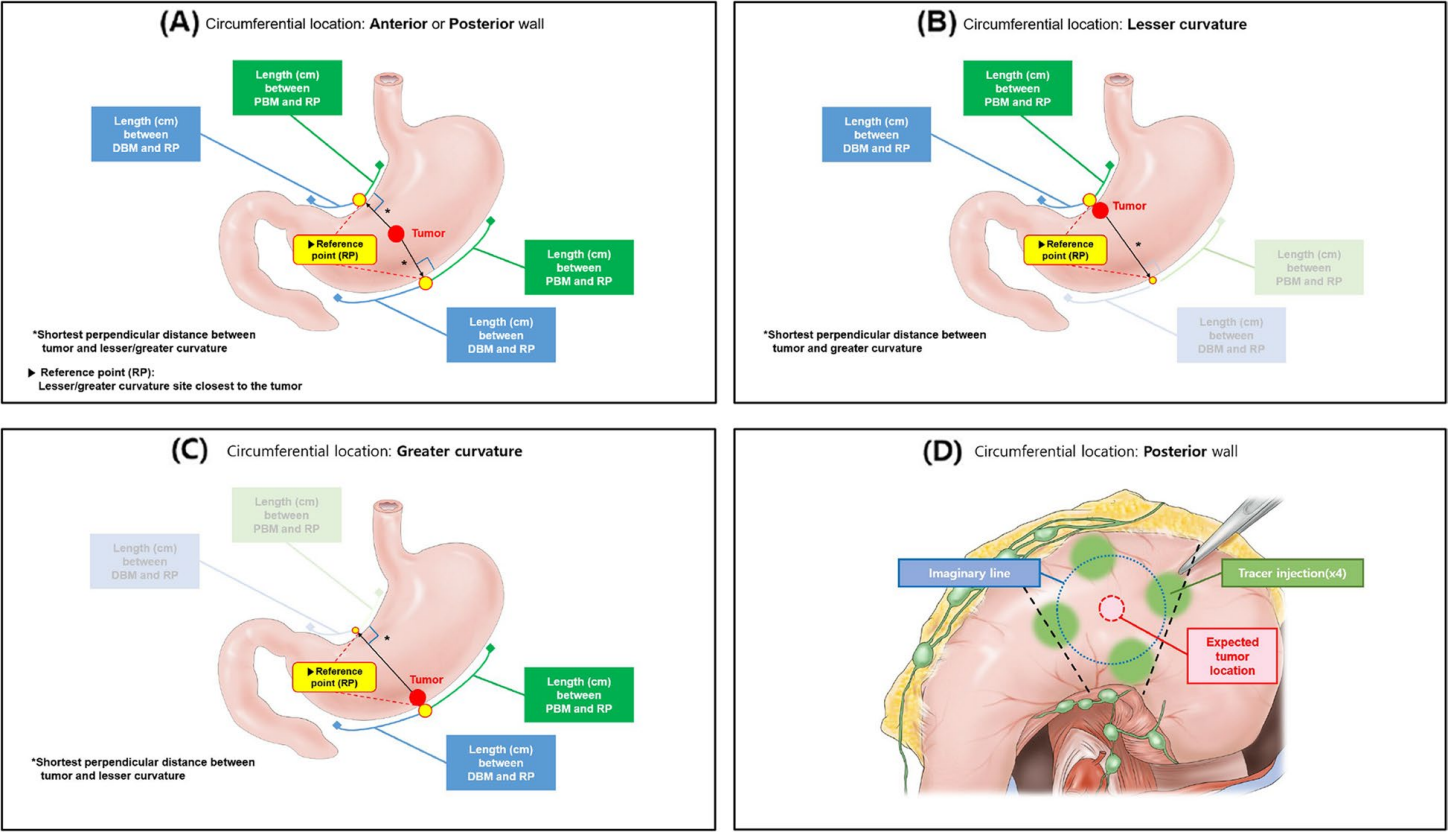
Evaluation of the proximal and distal basin margins according to circumferential location of the tumor. When the tumor was located at the anterior or posterior wall (**A**), the reference point was defined at the lesser or greater curvature site that was closest to the tumor, respectively. The lengths between the proximal/distal basin margin and reference point at the lesser/greater curvature were measured. When the tumor was located at the lesser curvature (**B**), the reference point was the center of the tumor. The lengths between the proximal/distal basin margin and reference point at the lesser curvature were measured. If the sentinel basin was not identified at the greater curvature side, we did not measure the length between the proximal/distal basin margin and the reference point at the greater curvature. When the tumor was located at the greater curvature (**C**), the reference point was the center of the tumor. The lengths between the proximal/distal basin margin and reference point at the greater curvature were measured. If the sentinel basin was not identified at the lesser curvature side, we did not measure the length between the proximal/distal basin margin and the reference point at the lesser curvature. When the tumor was located at the posterior wall and difficult to localize after immediate tracer injection, the tumor location was predicted after partial omentectomy with dissection of the greater curvature (**D**). Otherwise, we re-confirmed the tumor location with intraoperative endoscopy, if possible DBM = distal basin margin; PBM = proximal basin margin; RP = reference point

The lengths between the proximal and distal SB margins and the reference points at the lesser and greater curvature sides of the stomach were measured. When the tumor was located at the anterior or posterior wall of the stomach, the points where the perpendicular distance from the tumor to the lesser and greater curvature sides of the stomach were the shortest, respectively, were the reference points. We measured the proximal and distal lengths with a laparoscopic ruler from the two reference points on the lesser and greater curvature side of the stomach (Fig. 2A). When the tumor was located on the lesser curvature side, the reference point was the center of the tumor at the lesser curvature of the stomach (Fig. 2B). The lengths between the proximal/distal basin margin and reference point at the lesser curvature side were measured. If the SB was not identified on the greater curvature side, we did not measure the length of SBs on that side. When the tumor was located on the greater curvature side, the reference point was the center of the tumor at the lesser curvature of the stomach (Fig. 2C). Additionally, when the tumor was located at the posterior wall and difficult to localize after injection of tracer during SNNS, the tracer injection sites were visualized after performing omental dissection at the greater curvature side, and the tumor location was predicted (Fig. 2D). Otherwise, we re-confirmed the tumor location with intraoperative endoscopy, if possible. After the lengths of proximal and distal basin margins were measured, the SBs containing SLNs (green, hot, basin nodes) were carefully dissected by laparoscopy and retrieved from the surgical field.

### Statistical analysis

Continuous variables were analyzed using the Mann–Whitney *U* or Kruskal–Wallis test. Statistical analyses were carried out using SPSS version 27.0 for Windows (SPSS, Chicago, IL). If the *p* value was less than 0.05, the statistical difference was defined as significant.

## Results

### Patient demographics

The demographics of 25 patients are shown in Table 1. The mean age was 61.1 years, and the patients were predominantly male. The mean ± standard deviation of operation time was 197.9 ± 39.7 min, and the sentinel study time was 109.4 ± 17.9 min. Most patients underwent gastric wedge resection (44.0%) or gastric segmental resection (48.0%) with SBD. However, two patients (8.0%) required conversion to distal gastrectomy due to identification of metastatic SLN by frozen section biopsy examination during surgery. The mean ± standard deviation tumor size was 2.9 ± 1.6 cm, and 92% of patients were finally diagnosed with stage I. The mean ± standard deviation of the number of SLNs was 12.8 ± 7.2. On the lesser curvature side, the lengths of the SB (cm [min–max]) were 3.7 cm [2.0–5.0] proximally and 3.0 cm [2.3–5.5] cm distally. On the greater curvature side, the lengths of the SB were 6.8 cm [3.5–11.0] proximally and 7.0 cm [3.8–9.5] distally.

**Table 1:** Patient demographics (*n* = 25)

Factors	Value
Age (years)	61.1 ± 10.0
Sex	
Male	16 (64.0%)
Female	9 (36.0%)
BMI (kg/m^2^)	23.6 ± 2.5
Operation time (min)	197.9 ± 39.7
Sentinel study time* (min)	109.4 ± 17.9
Operation type	
LGWR with SBD	11 (44.0%)
LGSR with SBD	12 (48.0%)
Conversion to laparoscopic distal gastrectomy^†^	2 (8.0%)
Tumor size (cm)	2.9 ± 1.6
TNM stage, AJCC 8th ed	
I	23 (92.0%)
II	2 (8.0%)
III	1 (4.0%)
Number of sentinel lymph nodes	12.8 ± 7.2
Length of sentinel basin	
Lesser curvature (cm)	
Proximal length of sentinel basin	3.7 [2.0–5.0]
Distal length of sentinel basin	3.0 [2.3–5.5]
Greater curvature (cm)	
Proximal length of sentinel basin	6.8 [3.5–11.0]
Distal length of sentinel basin	7.0 [3.8–9.5]

*BMI* = body mass index, *LGWR* = laparoscopic gastric wedge resection, *SBD* = sentinel basin dissection, *LGSR* = laparoscopic gastric segmental resection, *AJCC* = American Joint Committee on Cancer

Values are presented as mean ± standard deviation, median [min–max] or number of patients (percentage)

^*^Time from the first injection of tracer to the identification of the frozen biopsy result for the sentinel lymph node

^†^Due to the identification of metastatic sentinel lymph node on frozen biopsy examination

### Mean lengths of proximal and distal sentinel basin

The mean lengths of proximal and distal SB margins from the reference points of patients categorized into subgroups by tumor characteristics are listed in Table 2. The lengths of the SB were similar between T1a (*n* = 14) and T1b or deeper (*n* = 11) tumors, except the length of the proximal SB at the greater curvature side in tumors that were T1a (8.0 cm [5.5–10.5]) and T1b or deeper (6.0 cm [3.5–11.0]). When the size of tumors was the same or less than 2 cm (*n* = 9), the lesser curvature side mean lengths of the proximal SB (2.9 cm [2.0–4.5] were shorter than those of the proximal SB (4.0 cm [2.0–5.0]), with tumor sizes longer than 2 cm (*n* = 16). When tumors were located at the low body or antrum of the stomach (*n* = 21) longitudinally, the median lengths of the proximal SB tended to be longer than those of tumors located at the high or mid-body of the stomach (*n* = 4), on both the greater and lesser curvature sides. When tumors were located at the lesser curvature side of the stomach (*n* = 7) circumferentially, the median lengths of the proximal SB (4.0 cm [2.4–5.0]) and distal SB (3.2 cm [2.5–4.0]) at the lesser curvature were longer than those of the proximal SB (2.0 cm) and distal SB (2.3 cm) of tumors located at the greater curvature when the SB was identified at the greater curvature side. When tumors were located at the greater curvature side of the stomach (*n* = 7), the mean lengths of the proximal SB (7.0 cm [5.5–8.5]) and distal SB (7.0 cm [4.0–8.0]) at the greater curvature were longer than those of the proximal SB (6.0 cm) and distal SB (5.5 cm) of tumors located at the lesser curvature when the SB was identified at the lesser curvature side. When tumors were located at the anterior wall of the stomach (*n* = 3) circumferentially, the median lengths of the SB were longer than those of tumors located at the posterior wall (*n* = 8) in both the greater and lesser curvature sides. The length of the proximal SB at the greater curvature side (10.0 cm [9.0–11.0]) was longest when tumors were located at the anterior wall of the stomach. When the number of SLNs in the total SB was 12 or less, the lengths of the proximal SB (6.0 cm [3.5–8.5]) and distal SB (5.5 cm [3.8–9.5]) at the greater curvature side were shorter than those of the proximal SB (8.0 cm [5.5–11.0]) and distal SB (7.5 cm [7.0–9.0]) when the number of SLNs in the total SB was 13 or more, and these differences were statistically significant (proximal SB *p* = 0.035, distal SB *p* = 0.006). However, the lengths of the proximal and distal SB were similar at the lesser curvature side regardless of the number of SLNs in the total SB. Similar results were obtained when comparing the lengths of SBs according to the number of SLNs in the main SB, which is the SB with the largest number of SLNs. There was a statistically significant difference in the distal SB length of the greater curvature side according to the number of SLNs in the main SB (≥ 10 versus ≤ 9, 7.3 cm [6.5–9.0] versus 5.5 cm [3.8–9.5], *p* = 0.027).

**Table 2:** Mean proximal and distal length of sentinel basin from the reference point categorized into subgroups

	No. of patien s (*n* = 25)	Lesser curvature				Greater curvature			
		PLSB (cm)	*P* value	DLSB (cm)	*P* value	PLSB (cm)	*P* value	DLSB (cm)	*P* value
Depth of tumor invasion			0.968		0.717		0.094		1.000
T1a	14	3.7 [2.0–4.5]		3.0 [2.3–5.5]		8.0 [5.5–10.5]		7.0 [4.0–9.5]	
≥ T1b	11	3.5 [2.0–5.0]		3.4 [2.3–4.0]		6.0 [3.5–11.0]		7.0 [3.8–9.0]	
Tumor size (cm)			0.244		0.639		0.375		0.791
≤ 2	9	2.9 [2.0–4.5]		3.0 [2.3–4.0]		6.0 [5.0–10.5]		7.0 [4.0–9.5]	
> 2	16	4.0 [2.0–5.0]		3.0 [2.3–5.5]		7.0 [3.5–11.0]		7.0 [3.8–9.0]	
Tumor location (longitudinal)			0.234		1.000		0.130		0.912
High or mid-body	4	2.7 [2.4–3.0]		3.1 [3.0–3.2]		5.5 [3.5–7.0]		7.0 [3.8–8.0]	
Low body or antrum	21	3.8 [2.0–5.0]		3.0 [2.3–5.5]		7.0 [5.0–11.0]		7.0 [4.0–9.5]	
Tumor location (circumferential)			0.146		0.204		0.194		0.300
Lesser curvature	7	4.0 [2.4–5.0]		3.2 [2.5–4.0]		6.0*		5.5*	
Greater curvature	7	2.0*		2.3*		7.0 [5.5–8.5]		7.0 [4.0–8.0]	
Anterior wall	3	4.0 [3.5–4.0]		4.0 [2.8–5.5]		10.0 [9.0–11.0]		8.3 [7.5–9.0]	
Posterior wall	8	2.9 [2.0–4.5]		3.0 [2.3–4.0]		6.3 [3.5–10.5]		5.8 [3.8–9.5]	
No. of SLNs in total SB			0.967		0.384		0.035		0.006
≤ 12	16	3.4 [2.0–5.0]		3.0 [2.3–5.5]		6.0 [3.5–8.5]		5.5 [3.8–9.5]	
≥ 13	9	3.7 [2.0–4.5]		3.2 [2.3–4.0]		8.0 [5.5–11.0]		7.5 [7.0–9.0]	
No. of SLNs in main SB^†^			0.898		0.210		0.101		0.027
≤ 9	15	3.5 [2.0–5.0]		3.0 [2.3–5.5]		6.0 [3.5–9.0]		5.5 [3.8–9.5]	
≥ 10	10	3.9 [2.0–4.5]		3.6 [2.3–4.0]		7.5 [5.5–11.0]		7.3 [6.5–9.0]	

*PLSB* = proximal length of sentinel basin; *DLSB* = distal length of sentinel basin; *SB* = sentinel basin; SLN = sentinel lymph node

Values are presented as median [min–max]

^*^Only the data from one patient is applicable

^†^Main SB is the SB with the largest number of SLNs among SB

### Frozen biopsy examination of sentinel lymph nodes and postoperative survey of recurrence

Among 25 patients, two showed macro-metastasis at the SLN by frozen section biopsy during laparoscopic SNNS. Both patients were to undergo gastric wedge resection with SBD; however, after identification of the SLN metastasis intraoperatively, both underwent conversion to laparoscopic conventional distal gastrectomy. One patient underwent total laparoscopic distal gastrectomy with Billroth II anastomosis and conventional radical lymphadenectomy, and the other underwent laparoscopic-assisted distal gastrectomy with Billroth I anastomosis and conventional radical lymphadenectomy. Twenty-three patients with no metastasis on the SLNs underwent laparoscopic SBD with stomach preserving surgery such as gastric wedge or segmental resection. Including the two cases of conversion to conventional radical gastrectomy, there was no occurrence of metachronous gastric cancer in the remnant stomach or recurrence at any other organ in the 25 patients enrolled in this study 5 years after surgery.

## Discussion

This study reported the length of the SB according to tumor characteristics of EGC based on data from a prospective RCT [6]. As far as we know, this is the first report investigating the length of the SB based on prospective clinical trial data.

After the effectiveness of the SLN biopsy was validated for treatment of specific cancers, SLN biopsy has become the standard procedure in the assessment of metastatic spread to the lymph node basin in breast cancer [12, 13] and malignant melanoma [14, 15]. Unlike conventional SBD, which dissects the entire lymph node and its surrounding tissue at a specific basin, SLN biopsy for breast cancer [16] and malignant melanoma [17] is usually performed by with manipulation of each radioactive or dyed lymph node. However, there are concerns about the low accuracy of SLN biopsy to manipulate lymph nodes during gastric cancer surgery, unlike SLN biopsy for breast cancer or melanoma, due to abundant perigastric fat tissue and the limitations of laparoscopic surgery. Therefore, in most trials, SBD was performed instead of SLN biopsy during gastric cancer surgery [18, 19]. In addition, the SBD procedure aims to perform localized lymph node dissection to minimize the possibility of recurrence even in cases with false-negative SLN biopsy results [20]. The SBD procedures that were reported recently are slightly different. In a previous Japanese study, the SBD procedure involved dissection of all lymph nodes including the SLNs in a particular lymph node station, which was objectively classified in the Japanese Classification of Gastric Carcinoma [21–23]. On the other hand, during the SENORITA trial, we removed only the basin after detecting the extent of the SB [8]. The SENORITA trial followed a prospective multicenter feasibility study prior to the multicenter phase III RCT [10]. This prior study showed the feasibility of laparoscopic SBD and demonstrated improved results in detecting metastatic lymph nodes; it had a 100% sensitivity rate, 100% false-negative rate, and 0% negative predictive value for SBD, which indicated little to no possibility of missed lymph node metastasis. Among these two SBD methods of the Japanese and SENORITA trials, there is no evidence for which is more accurate and appropriate for SBD. When the metastasis of the SLN can be found without tracer injection during surgery, the oncologic outcomes tend to be good.

To perform stomach preserving surgery, certain steps in laparoscopic SBD are essential. As previously mentioned, after endoscopic injection of Tc99m-HAS and ICG in the SENORITA trial, tracing was required with a laparoscopy camera and a laparoscopic handheld gamma probe. According to the protocol of SBD procedures, we needed to wait 15 min after endoscopic tracer injection to detect the extent of the SBs [8]. In addition, after the SBD procedure, an immediate frozen section biopsy examination by pathologists was needed for stomach preservation [6, 9]. In summary, laparoscopic SNNS for stomach preserving surgery requires additional procedures with longer operation time, endoscopic and pathologic examinations during surgery, and more surgical instruments and manpower compared to the conventional laparoscopic radical gastrectomy for gastric cancer. In addition, there might be subtle radiation exposure to the patient and surgeons when using Tc99m-HSA. In this study, we made suggestion that the SBD procedure can be performed without the use of a tracer. The advantage of SBD without using the tracers is that it not only shortens the operation time (at least more than 15 min), but also reduces medical costs and manpower. In addition, by omitting tracer-related procedures, exposure to radioactive isotopes can be avoided, and there is no need to undergo radiation safety management. In our previous report, the median operation time was significantly longer in laparoscopic SNNS (195.0 min) compared to laparoscopic conventional gastrectomy (180.0 min) in the per-protocol analysis (*P* < 0.001) [7]. This indicates that the procedure is complicated and requires much more preparation for the current laparoscopic stomach preserving SNNS. In Table 1, the mean ± standard deviation value of sentinel study time was 109.4 ± 17.9 min. We describe the sentinel study time as the time from the first injection of tracer to identification of the frozen biopsy result for the SLNs in this study. The steps of laparoscopic SBD were as follows [9]: (A) endoscopic injection of Tc99m-HSA with ICG and a laparoscopic view after tracer injection. (B) laparoscopic sentinel basin node detection along the greater and lesser curvatures of the stomach. (C) Surgical clip application for marking the extent of laparoscopic SBD in the greater and lesser curvatures of the stomach. (D) Laparoscopic SBD along the greater and lesser curvatures of the stomach. (E) Completion of laparoscopic SBD along the greater and lesser curvatures of the stomach. Among these steps, we proposed skipping procedures (A) and (B). To become a generalized procedure, a simpler and more convenient method for SBD should be developed.

This study was based on the hypothesis that the SBD procedure based on the location of the tumor will be similar among patients since the extent of sentinel lymphatic flow is based on anatomical location. If there is no need for tracer injection and SB detection, the laparoscopic SBD procedure, which can be called localized regional lymphadenectomy around the stomach, will be performed more conveniently, and the duration of the SBD procedure will be reduced. In addition, there is no need for tracers using radioactive isotopes, which might cause radiation exposure to patients and clinicians, and no need to prepare a fluorescence laparoscopic camera to detect an ICG tracer. In this study, two of the 25 patients were diagnosed with SLN metastasis at intraoperative frozen section biopsy. If the extent of SB can be predicted in advance during surgery, the metastatic SLN can be identified by SBD with frozen section biopsy, without need for tracer injection and detection. Sequentially, conversion to conventional radical gastrectomy can proceed after confirmation of metastasis at SLNs by intraoperative frozen section biopsy.

In addition, the function of the stomach can be maintained after performing SBD, which is an advantage over conventional radical gastrectomy. Based on the protocol of the SENORITA trial, patients with a gastric tumor at least 2 cm from the cardia or pylorus were included to preserve the function of the gastric sphincters. Therefore, we could have preserved the stomach function including those of the proximal and distal sphincters [8]. According to the results of the SENORITA trial, function preserving surgery was possible after SLNBD, and the quality of life of patients who received that surgery was improved compared to those who underwent conventional gastrectomy [6]. The data from this prospective study suggest clinical application of regional lymphadenectomy with intraoperative evaluation of the SLN, which can be called D1-lymphadenectomy for EGC. With the length of SBD, we can simplify the steps of laparoscopic stomach preserving SNNS. However, when anatomical landmarks such as the lower esophageal sphincter, pyloric sphincter and vagus nerve, which are important for function preserving surgery, overlap with the extent of SB length, and if there is potential for damage of these structures, surgeons are recommended to perform the conventional gastrectomy.

The limitations of this study and hypothesis are as follows. First, this study enrolled a small number of patients and this measured SB length may not be currently applicable. The oncological safety is a concern due to differences in SB length values among a small number of enrolled patients and lack of research on lymph node station 8a. In a previous study concluded that when SLN was detected at station 8a, the other SLNs at stations 5, 6, and 7 should be confirmed by pathologic examination and function preserving surgery should be re-considered [24]. Therefore, routine measurements of SB length and research on SLN at station 8a are needed to make a generalized standard when performing SBD in the future. Second, the extent or length of the SB might differ by tracer injection site around the tumor based on endoscopist. The need for a more accurate protocol for tracer injection sites should be considered. Also, the indications for patients who can apply to undergo SNNS without tracer should be strict. The operator must select appropriately between SNNS or conventional gastrectomy when patients are diagnosed with metachronous cancers, large size cancer, and cancer at ambiguous location. Third, although we perform SBD without a tracer, a time-consuming intraoperative back table SLN distinction procedure and frozen section biopsy examination for SLNs were needed. In addition, we needed a procedure to localize the tumor during laparoscopic surgery, such as intraoperative endoscopy. In the SENORITA trial, surgeons started the SBD procedure at 15 min after tracer injection. Therefore, this could save at least 15 min when we perform SBD without the tracer injection. Further research on SBD without the use of tracer is needed for reducing additional sentinel lymph node study time. Last, it is possible for surgeons to describe the longitudinal location of the tumor based on the site of the gastric angle. On the other hand, it is difficult to describe the exact circumferential location of the tumor due to the lack of gastric landmarks. Even when the circumferential location of the tumor is ambiguous, we suggest that the surgeons dissect the SBs at both the lesser and greater curvature sides of the stomach from the reference point to proximal or distal SB margins. Considering the blood supply at the SBD site around the stomach, either a gastric wedge or segmental resection is recommended instead of endoscopic submucosal dissection due to the risk of delayed perforation by reduced blood supply [25]. The value of application for the SNNS without tracer which we suggest can be dampened because of the limited number of enrolled cases, the anatomical variance of stomach, and the potential bias of the measurement of SBs length in this study. Nevertheless, the present prospectively collected data on the extent of SBs could be a basis for simplifying laparoscopic SNNS.

We reported lengths of 3.7 cm [2.0–5.0] proximally and 3.0 cm [2.3–5.5] cm distally of the SB along the LC and lengths of 6.8 cm [3.5–11.0] proximally and 7.0 cm [3.8–9.5] cm distally for the SB along the GC of the stomach. The length of the SB presented in this study might be a reference for identifying extent of SB without the use of tracers. Application of these methods may help to simplify the complicated and time-consuming procedures of laparoscopic SNNS for stomach preservation in EGC. Currently, with the measured SBD length alone, SNNS cannot be performed without the tracer. This is a proposal of a simple way to perform SNNS without the tracer, and showed the reference of SBD length with small number of patients. In the future when performing SNNS with the tracer, we need to research to make indications for patients who can undergo SNNS without tracer and investigate the relationship between tracer flow, recurrence pattern and SBD length to confirm if there are problems with oncological safety.

## Data Availability

The datasets generated and/or analyzed during the current study are available from the corresponding author upon reasonable request.
